# Brief Interventions for Hazardous and Harmful Alcohol Consumption in Accident and Emergency Departments

**DOI:** 10.3389/fpsyt.2014.00152

**Published:** 2014-11-03

**Authors:** Marcin Wojnar, Andrzej Jakubczyk

**Affiliations:** ^1^Department of Psychiatry, Medical University of Warsaw, Warsaw, Poland; ^2^Department of Psychiatry, University of Michigan, Ann Arbor, MI, USA

**Keywords:** emergency department, brief intervention, alcohol, implementation, effectiveness

## Abstract

The prevalence of alcohol abuse among patients treated in accident and emergency departments (A&E) is considered as substantial. This paper is a narrative review of studies investigating the effectiveness of brief interventions (BI) for hazardous and harmful alcohol consumption in A&E. A&E departments in hospitals (and other health care infrastructures) are commonly the place where serious consequences of alcohol drinking are seen and need to be tackled, supporting the suggested theoretical usefulness of delivering BI in this environment. Available research shows that BI may be considered a valuable technique for dealing with alcohol-related problems. However, it is suggested that the usefulness of BI may depend significantly on the target population to be dealt with. BI have proved to be beneficial for male individuals and those patients who do not abuse other psychoactive substances. In contrast, evidence indicates that BI in A&E settings are not effective at all when dealing with men admitted as a consequence of a violence-related event. In addition, some studies were unable to confirm the effectiveness of BI in female population, in emergency setting. Studies investigating the association between drinking patterns and the effectiveness of BI also present inconsistent results. Most studies assessing the effectiveness of BI in A&E settings only adopted a short perspective (looking at the impact up to a maximum of 12 months after the BI was delivered). When assessing the effects of BI, both the amount of alcohol consumed and expected reductions in alcohol consequences, such as injuries, can be taken into account. Evidence on the implementation of brief intervention in emergency departments remains inconclusive as to whether there are clear benefits. A variety of outcome measures and assessing procedures were used in the different studies, which have investigated this topic.

## Introduction

Accident and emergency departments (A&E) can be defined as a medical treatment setting specialized in acute care of patients who are admitted presenting rapid symptoms and without prior appointment. The prevalence of alcohol abuse among patients treated (or least diagnosed) in this setting is considered to be substantial. Not surprisingly, alcohol is regarded as one of the leading causes of car accidents (1) as well as injuries in general (2). According to different studies, up to 77% (2, 3) of subjects admitted in A&E presented an alcohol-related injury, with about 30% of patients drinking alcohol above recommended levels (4), and between 6 and 22% (2, 3) meeting the criteria of alcohol dependence according to DSM-IV (5). It has been shown that injuries (which are responsible for about 40% of visits to A&E) are especially associated with alcohol drinking. A recent systematic review by Zerhouni et al. (6) has shown that, compared to uninjured patients, individuals with injuries admitted into A&E have a significantly higher probability of presenting elevated blood alcohol levels. Furthermore, in comparison to non-injured individuals entering A&E, injured individuals more frequently report having drunk alcohol during the 6-h preceding the fatal event and suffer from drinking-related consequences that adversely affect their social life. Notably, a recent meta-analysis (7) has shown that compared with general population, male subjects with alcohol-use disorders had more than 6.5-fold higher risk for mortality by injury in 10 years follow-up.

The above mentioned numbers regarding prevalence of alcohol-related problems in A&E exceed the general frequency of dealing with patients with problem drinking that occurs in other medical settings (8). However, they also show that most of the victims of alcohol-related health problems admitted to A&E are not alcohol-dependent individuals according to DSM-IV, but rather subjects drinking alcohol in a risky or harmful way. Therefore, actions aimed at reducing the amount of alcohol consumed but not requiring absolute abstinence such as SBIRT (*screening, brief intervention, and referral to treatment*) programs may be considered particularly useful.

The drinking pattern of special concern in A&E departments would probably be binge drinking (defined as consuming four or more standard drinks for females and five or more standard drinks for males on one occasion; with one standard drink containing 10 g of pure ethanol), which has been identified as particularly risky, leading to injuries and general health consequences (9, 10). Moreover, A&E is usually the place where individuals are confronted for the first time with serious consequences of their own or others’ alcohol drinking. Therefore, the usefulness of brief interventions (BI) in this setting is strongly supported.

The aim of this paper was to summarize the available evidence on the effectiveness of BI in A&E departments, as well as the effectiveness of specific BI implementation strategies that have been used in this setting. In order to do so, the Medline database has been searched using the following terms: “brief intervention,” “alcohol,” “emergency department,” “emergency room,” “accident and emergency room,” “alcohol drinking,” “harmful drinking,” and “screening.” All papers included in this review were published during the last decade, as to provide a summary of the most recent research results. References listed in all selected articles were additionally searched.

## Efficacy of BI in Accident and Emergency Departments

A systematic review performed by Nilsen et al. (11) showed that a positive effect of BI delivered in A&E on the level of alcohol drinking or the frequency of injuries was observed in 11 out of 12 studies. In addition, more intensive interventions were shown to yield more positive effects. The positive effects were observed in alcohol intake, risky drinking practices, alcohol-related negative consequences, and injury frequency, although in different studies different outcomes were measured and not in all of these outcome measures a positive effect was observed. In more recent randomized controlled trials, the positive effect of BI has been shown to reduce all outcomes: number of drinking days, amount of alcohol drunk on a single occasion, as well as the negative consequences of drinking (12). Notably, the study by Cherpitel et al. provides evidence for effectiveness of BI itself not just as an assessment reactivity (reactivity to the results of questionnaires evaluating amount of alcohol drunk and consequences of drinking), which has been raised as a potential mechanism of BI efficacy (12). In addition, Drummond et al. (13) showed that, contrary to the conclusions of the review by Nilsen et al., more intensive clinical interventions do not add significant benefits to very simple and short interventions. Importantly, BI directed toward subjects in the mild range of drinking severity have been shown to be significantly less effective compared to BI used in individuals within the moderate to heavy range of drinking (12).

In available research studies on BI in A&E departments, BI were shown to be effective in the short-term (with a follow-up measure up to 12 months after the BI took place). Most of these studies have assessed effectiveness in 12 months of observation only (14–17), whereas the few projects, which followed subjects for a longer period of time, did not confirm long-term effectiveness. On the other hand, in a study by Gwaltney et al. (18) the effects of BI did not emerge immediately after an initial session (evaluation after 1 month), but became visible later – at 3- and 6-months follow-up visits. In a study by Woolard et al. (19), a reduction of binge drinking days occurred and persisted also during 12 months of observation after delivering BI; however, a significant decrease in the consequences of drinking (such as injuries) was not observed when the BI group was compared to controls. However, results of many studies, also confirmed by meta-analyses (11, 20), suggest the opposite association – that BI reduces negative consequences of alcohol rather than the amount of alcohol consumed.

The analysis of the literature shows that the results of the studies on the effectiveness of BI in accident and emergency departments, although in most cases encouraging, remain inconsistent. D’Onofrio et al. (21) described no differences in effectiveness between emergency practitioner-performed Brief Negotiation Interview and usual discharge instructions in terms of either alcohol-use or alcohol-related negative consequences. As previously described, numerous studies showed that the experience of being injured and having to be attended in an emergency department by itself may provide enough motivation for reducing drinking, without any alcohol-related intervention taking place (11). Also, it has been suggested that the environment of A&E with its chaos, hurry, and quick decisions is not a context offering a desirable atmosphere enabling reflection for change (11). Finally, in the randomized controlled clinical trial performed by Daeppen et al. (22), no effect of BI delivered in A&E on alcohol drinking outcomes during the follow-up was observed.

Interestingly, the use of SBIRT techniques and questionnaires in a group of non-risky drinkers from emergency settings has been shown to lead to *increases* in the amount of alcohol drunk (23). The authors suggested as an explanation that these non-risky drinking subjects might have felt to be low consumers and at “safe levels” according to the presented thresholds, which emphasizes the significance of screening in this procedure.

## Gender Perspective

Like in other settings (24, 25), results of the studies considering gender differences in the efficacy of BI in accident and emergency departments are inconsistent and conflicting. In a study by Choo et al. (26) BI turned out not to be effective at all in female participants and successful in men, but only for those without a history of involvement in violence. Also in a study by Woodruff et al. (27), men were more likely than women to benefit from BI, However, in another study, no differences between genders were reported (28), whereas, in a study by Blow et al. (16), younger adult women (ages 19–22 years) were most likely to decrease their heavy episodic drinking after receiving brief advice. Despite these contradicting findings, the general trend is for men to be more prone to benefit from BI in A&E settings. This observation is consistent with research findings showing that alcohol misuse is commonly a primary problem in male individuals, while in women alcohol drinking is often associated with other psychiatric conditions (personality, depressive, or anxiety disorders) (29). Therefore, from this perspective, interventions in women may be more effective when aimed at psychiatric symptoms rather than drinking itself. On the other hand, in male individuals interventions directed on drinking itself may appear to be the most appropriate, reasonable strategy.

## Brief Interventions in Young Individuals

It has been suggested that emergency departments are especially suitable for interventions of a preventive kind as the age of patients treated in A&E is lower than in any other medical setting, thus allowing identification of harmful and risky behaviors at early stages. The results of studies conducted in young individuals are in accordance with data concerning older patients. Two systematic reviews of literature concerning the effectiveness of BI in A&E in youngsters and college drinkers show that most of the studies confirm a positive effect of BI in alcohol drinking (30, 31). BI turned out to be effective in reducing alcohol intake and risky behaviors associated with drinking [including aggression (32)], although these effects were measured in just a short-term (up to 1 year) perspective (30, 31, 33). Also, similarly to adult individuals, the female gender was shown to be associated with significantly weaker effects of BI in adolescents (34), and computer-assisted SBIRT procedures were shown to be as effective as those conducted face-to-face (33). However, due to numerous inconsistencies in the results of previous research studies (33, 35), clear conclusions concerning the usefulness of delivering BI to the young population in emergency settings cannot be drawn.

## Patients’ Characteristics

Few studies have investigated the psychological factors contributing to the success (or lack of success) of BI in emergency departments. The small number of studies aimed at more sophisticated elaboration of the procedure is, however, consistent with the core idea of brief intervention, which is that it has to be brief, easy to implement, and not time-consuming. Designing a study assessing psychological factors and investigating detailed mechanisms of BI effectiveness would probably mean that the whole procedure would no longer be brief.

Brief interventions have been shown to be particularly effective in individuals who attribute their injury to alcohol (36, 37), suggesting that one of the major aims of BI in accident and emergency departments may be to identify the link between alcohol and injury. As previously mentioned (see Gender Perspective), BI were reported to be ineffective in individuals involved in violent actions (26). The largest reduction in drinking following discharge from A&E without receiving a BI was observed in subjects characterized by a history of alcohol-related accidents and injuries, and more severe consequences of drinking in general (37). Most likely, this was the group with highest motivation and readiness for change, which has been identified as one of the main factors diminishing the effectiveness of brief intervention (38).

As mentioned previously, a history of involvement in violence (26) and comorbid misuse of other substances (than alcohol) (27) have been shown to decrease the effectiveness of BI in A&E departments. These observations emphasize the plausible association between the level of psychopathology (e.g., personality disorders) and efficacy of BI in accident and emergency departments. This issue remains a possible objective for further research, although this idea may be challenged by the concept of BI as a short, not complicated, and easy to administer intervention.

## Implementation of Brief Interventions in the Emergency Setting

As shown above, the emergency setting seems to be an appropriate place to introduce BI for alcohol drinking, both from a theoretical and a clinical perspective, whereas the efficacy of BI in A&E has been shown by the results of most of the studies. It has been emphasized that the moment directly after an accident or admission to A&E may be considered the most “teachable” one. Moreover, in most of the studies reduction of drinking was even observed in control groups not receiving BI (11), making it reasonable to assume that the injury and admission to A&E by itself constitute a motivation for change in drinking habits, and thus a favorable moment to take advantage for delivering BI. Notably, the implementation of BI in accident and emergency department has also been recommended in official guidelines for alcohol prevention (39–41). However, the specificity of A&E departments includes brevity of contact with patients, overloading of the staff, and engaging in numerous activities that may be considered more important and more directly associated with life-saving approaches that remain the core of A&E functioning. In addition, a lack of adequate training has been suggested as a significant barrier in implementing BI in the emergency setting (42, 43).

This clear discrepancy between the needs and capabilities has been confirmed in numerous studies showing that less than one-third of accident and emergency departments offers BI for alcohol drinking by trained personnel (42). Such surprisingly low prevalence of BI implementation stimulated studies aimed at identifying barriers and facilitators of SBIRT use in the emergency setting. Among possible facilitators, presence of official guidelines and health policy (44), as well as use of computer-assisted screening and brief intervention procedures (45, 46) were emphasized. In addition, during the introduction of a screening procedure into everyday routine practice (47), a positive change in attitudes toward screening and BI of A&E staff was observed, as they reported to experience that the procedure worked well and that patients were willing to cooperate.

## Different Ways of Implementing Brief Intervention

Taking into account the barriers and facilitators identified in previous research for implementing BI in emergency settings, a few recent research studies focused on the possibility of using a computer-assisted SBIRT procedure in A&E. The results of these studies show that the use of such technological supports substantially increases screening rates (even up to 89–97%) (45, 46), but the outcomes in terms of effectiveness remain inconsistent (11). Therefore, more research is needed to establish knowledge about possible benefits of technologically modified SBIRT procedures.

Other research analyzing the kinds of intervention performed, showed that individuals for whom alcohol was not a factor involved in the injury or cause of admission, although they presented symptoms of risky or harmful alcohol use justifying the delivery of BI benefited from a counselor-guided intervention (consideration of the risks related to their alcohol use), whereas subjects already experiencing previous consequences of alcohol use did not show added benefit from the counselor intervention compared to receiving the feedback report only (28).

Telephone-applied BI delivered orally decreased impaired driving and alcohol-related injuries in patients discharged from A&E with greatest effects for those with more severe alcohol drinking (48, 49). However, this type of intervention was not shown to be effective in terms of change in alcohol consumption and other alcohol-related consequences (49). In a recent multisite randomized clinical trial, brief intervention using personalized feedback delivered before the subject was discharged from A&E and followed by a telephone booster once discharged, was shown to be the most effective way of reducing alcohol drinking in A&E patients (50).

## Conclusion

Available research studies show that BI may be considered a useful technique for dealing with alcohol problems in A&E departments. However, it is suggested that the usefulness of BI may depend significantly on the population that it is offered to. The effects of BI can be measured both in terms of the amount of alcohol consumed and in terms of expected reductions in alcohol consequences, such as injuries, and therefore, different outcome measures were taken into consideration and a variety of assessing procedures were used in the studies addressing this topic. In addition, different methods of BI implementation were assessed, hampering the comparison between results. It is also important to consider that the number of research studies on the effectiveness of BI in A&E settings is still relatively small, while the most important methodological limitation of such studies consists in the fact that in most of them BI effectiveness was assessed in a short (up to 12 months) perspective. Although, it may seem challenging for a *brief* intervention to result in significant long-term effects, it would be useful to examine their effectiveness in longitudinal research studies designed for long-term observation, also providing more insight as to whether BI delivered in A&E departments have a significantly greater impact in reducing drinking and alcohol-related harm than the effect of being admitted into A&E after an alcohol-related event in itself.

## Conflict of Interest Statement

The authors declare that the research was conducted in the absence of any commercial or financial relationships that could be construed as a potential conflict of interest.
